# Food Administration and Not Genetic Variants Causes Pharmacokinetic Variability of Tadalafil and Finasteride

**DOI:** 10.3390/jpm13111566

**Published:** 2023-10-31

**Authors:** Gonzalo Villapalos-García, Pablo Zubiaur, Cristina Marián-Revilla, Paula Soria-Chacartegui, Marcos Navares-Gómez, Gina Mejía-Abril, Andrea Rodríguez-Lopez, Eva González-Iglesias, Samuel Martín-Vílchez, Manuel Román, Dolores Ochoa, Francisco Abad-Santos

**Affiliations:** 1 Clinical Pharmacology Department, School of Medicine, Hospital Universitario de La Princesa, Instituto Teófilo Hernando, Instituto de Investigación Sanitaria La Princesa (IP), Universidad Autónoma de Madrid, 28006 Madrid, Spain; g.villapalos@salud.madrid.org (G.V.-G.);; 2 Centro de Investigación Biomédica en Red de Enfermedades Hepáticas y Digestivas (CIBERehd), Instituto de Salud Carlos III, 28029 Madrid, Spain

**Keywords:** tadalafil, finasteride, food administration, pharmacogenetics

## Abstract

Tadalafil and finasteride are used in combination for the management of benign prostatic hyperplasia (BPH). Genetic variations in genes involved in the metabolism and transport of tadalafil or finasteride (i.e., pharmacogenes) could affect their pharmacokinetic processes altering their drug exposure, efficacy, and toxicity. The main objective of this study was to investigate the effects of variants in pharmacogenes on the pharmacokinetics of tadalafil and finasteride. An exploratory candidate gene study involving 120 variants in 33 genes was performed with 66 male healthy volunteers from two bioequivalence clinical trials after administration of tadalafil/finasteride 5 mg/5 mg under fed or fasting conditions. Afterwards, a confirmatory study was conducted with 189 male and female volunteers receiving tadalafil 20 mg formulations in seven additional bioequivalence clinical trials. Regarding tadalafil, fed volunteers showed higher area in the time-concentration curve (AUC_∞_), maximum plasma concentration (C_max_), and time to reach C_max_ (t_max_) compared to fasting volunteers; male volunteers also showed higher AUC_∞_ and C_max_ compared to female volunteers. Furthermore, fed volunteers presented higher finasteride AUC_∞_, C_max_ and t_max_ compared to fasting individuals. Variants in *ABCC3*, *CYP1A2*, *CES1*, *NUDT15*, *SLC22A1/A2* and *UGT2B10* were nominally associated with pharmacokinetic variation in tadalafil and/or finasteride but did not remain significant after correction for multiple comparisons. Genetic variation did not demonstrate to clinically impact on the pharmacokinetics of finasteride and tadalafil; however, additional studies with larger sample sizes are needed to assess the effect of rare variants, such as CYP3A4*20 or *22, on tadalafil and finasteride pharmacokinetics.

## 1. Introduction

Benign Prostatic Hyperplasia (BPH) is a disease characterized by the proliferation of the epithelial and the smooth muscle layers of the prostate, resulting in non-malignant growth of prostate tissue [1]. BPH arises from the loss of homeostasis between cellular proliferation and cell death, resulting in an imbalance favoring cellular proliferation. The prevalence of BPH is approximately 10% for 30-year-old men, increasing from 80% to 90% for those over 70 years of age [2]. BPH is frequently associated with Lower Urinary Tract symptoms (LUTS), which include symptoms related to obstruction (i.e., weak urine flow and incomplete emptying) and bladder storage problems (i.e., frequency of urination, urgency and nocturia) [3]. Furthermore, Erectile Dysfunction (ED) is a common sexual dysfunction characterized by the inability to achieve and/or maintain an erection firm enough to permit sexual intercourse [4]. ED can significantly worsen the quality of life of patients and their partners; it may be an early manifestation of generalized endothelial dysfunction and other forms of cardiovascular disease [5]. This condition is linked to several risk factors, including increasing age, depression, obesity, diabetes mellitus, hypertension and cardiovascular disease [5]. Some studies affirm that there is a strong correlation between LUTS secondary to BPH (LUTS/BPH) and ED. In fact, medical treatments for LUTS/BPH can significantly influence sexual function [6]. Although ED and LUTS/BPH are not often life-threatening conditions, their impact on quality of life can be significant and should not be underestimated.

Patients with moderate to severe symptoms may need pharmacological treatment. The two most frequently prescribed groups of drugs for BPH management are α-1 Adrenergic Receptor Antagonists (α1-ARAs) and 5-α Reductase Inhibitors (5-ARIs) [7,8]. On the one hand, α1-ARAs mediate smooth muscle relaxation and vasodilation by blocking the sympathetic fibers in the prostate and bladder, addressing the dynamic component of BPH and thus improving urinary-flow rate [2,3,9]. This family includes non-selective α-1 adrenergic blockers, such as doxazosin and terazosin, and selective α-1 adrenergic blockers, such as alfuzosin and tamsulosin [2,3]. On the other hand, 5-ARIs block the enzymatic activity of 5-α reductase, which is involved in the conversion of testosterone to dihydrotestosterone (DHT) [2,3]. Since androgens are involved in prostatic growth and enlargement, a decrease in serum testosterone and DHT would delay prostate enlargement and reduce prostatic volume (PV) [2,7,10]. Current available 5-α reductase inhibitors are finasteride and dutasteride [2,3]. Additionally, phosphodiesterase-5 inhibitors (PDE5-Is), which were initially approved by the FDA and EMA for the treatment of ED, also improve LUTS [3]. PDE5-Is, namely sildenafil and tadalafil, are similar to cyclic guanosine monophosphate (cGMP) in structure; thus, PED5-Is can bind to PDE5 competitively and inhibit cGMP hydrolysis, leading to prostate and urethra smooth muscle relaxation [3,11,12]. PDE5 inhibition may also provide improved blood flow to the lower urinary tract and reduce intraprostatic inflammation [3,12].

Previous research confirmed the effectiveness of combination therapy over monotherapy [3,13]. Combining the available pharmacological classes in the treatment of LUTS/BPH allows the patient to derive the benefits from each class, thus potentially maximizing treatment response. The most common combination is 5-ARI + ARA (i.e., dutasteride/tamsulosin) due to its efficacy in improving symptoms and preventing disease progression [14]. Nonetheless, in this study, tadalafil and finasteride combination was analyzed. PDE-5 inhibitors with 5-ARIs offer a novel therapy capable of producing positive treatment results comparable in efficacy with α-blocker and 5-ARI combinatorial therapy [15]. The combination therapy with tadalafil and finasteride reported an improvement in BPH/LUTS, as well as improved erectile function [16]. Finasteride reduces prostate volume, while tadalafil mediates lower urinary tract smooth muscle relaxation via PDE5 inhibition [15].

Regarding tadalafil pharmacokinetics, the mean area under the plasma concentration-time curve (AUC) following a 20 mg oral dose administration is 8066 ng·h/mL, the maximum plasma concentration (C_max_) is 378 ng/mL, and the time to reach C_max_ (t_max_) is approximately 2 h [15,17]. Tadalafil shows a lower systemic clearance compared to other PDE-5 inhibitors, with an elimination half-life (t_1/2_) of 17.5 h in healthy men and 21.6 h in the elderly [18]. The prolonged t_1/2_ of tadalafil is related to a volume of distribution (V_d_) of 60–70 L, slow hepatic clearance, and approximately 80% bioavailability [19]. The cytochrome P450 (CYP) 3A4 (CYP3A4) enzyme mainly catalyses the oxidative metabolism of tadalafil to a catechol metabolite, which is extensively methylated and glucuronidated to methylcatechol glucuronide metabolites [15]. Oral tadalafil is predominantly excreted as inactive metabolites through feces (61%) and, to a lesser extent, through urine (36%).

Regarding finasteride pharmacokinetics, after a 5 mg single-dose administration, the mean AUC was 267–288 ng·h/mL, the C_max_ was 38 ng/mL, and the t_max_ was approximately 2 h [20]. In addition, finasteride shows an absolute oral bioavailability of 80%, and it is highly plasma protein bound (90%) [15,21]. The drug is widely distributed into peripheral tissues, including the prostate gland, with a steady-state volume of distribution (V_d_) of 76 L. Finasteride is extensively metabolized in the liver through oxidative mechanisms by CYP450, specifically by the 3A4 isoenzyme, to essentially inactive metabolites, including ω-hydroxyfinasteride and finasteride-ω-oic acid, which are eliminated mainly through feces and bile (75%), but also through urine (25%) [15,21,22]. The finasteride total plasma clearance is reported to be 9.9 L/h (from a 4.2–16.9 L/h range), and its t_1/2_ is approximately 9 h [15].

One of the most effective alternatives for the adjustment and personalization of pharmacological treatment is the implementation of pharmacogenetic markers. Multiple therapies such as statins, fluoropyrimidines, etc., benefit from pharmacogenetic dose adjustment algorithms that allow more effective and safer treatments, i.e., reducing the incidence of adverse drug reactions (ADRs) [23,24]. However, there are no clinical guidelines available for adjusting the dosage of tadalafil or finasteride published by any international institutions or regulatory agency. Nonetheless, two studies identified associations between variants in different genes (i.e., *CYP3A4*, *SRD5A2*) with variations in finasteride serum concentrations [25] or drug’s affinity for its target [26]. On the contrary, no pharmacogenetic information is available for tadalafil.

Therefore, the main objective of this study was to identify the potential genetic variants related to altered pharmacokinetic processes of tadalafil and finasteride. As secondary objectives, the influence of feeding conditions, biogeographic group of origin, and sex were studied. For this purpose, pharmacokinetic data obtained in two bioequivalence clinical trials were analyzed according to genetic variants located in relevant pharmacogenes and other relevant demographic parameters such as weight, age, or biogeographic group of origin. Sixty-six healthy volunteers consented to this candidate-gene study for both tadalafil and finasteride. Subsequently, significant variables from the exploratory analysis were analyzed in seven confirmatory bioequivalence clinical trials for tadalafil, involving 189 volunteers.

## 2. Material and Methods

### 2.1. Study Area and Demographic Characteristics

The study population involved healthy males (in clinical trials 1, 2, 8, and 9) and males and females (in clinical trials 3, 4, 5, 6, and 7) aged from 18 to 55 years old who participated in nine bioequivalence clinical trials in Clinical Trial Unit of Hospital Universitario de La Princesa (UECHUP).

### 2.2. Ethical Approval

The protocol and informed consent for all clinical trials were reviewed and approved by the Independent Ethics Committee on Clinical Research (IECCR) of Hospital Universitario de La Princesa, and by the Agencia Española de Medicamentos y Productos Sanitarios (AEMPS). In addition, they were performed in accordance with the guidelines of the International Conference on Harmonization for Good Clinical Practice (ICH-GCP), current Spanish legislation, and the Revised Declaration of Helsinki [27,28]. This pharmacogenetic study was approved by IECCR of Hospital Universitario de La Princesa (registry number 4176, 9 July 2020).

### 2.3. Criteria and Conditions

The inclusion criteria in clinical trials 1 and 2 included male volunteers who were either surgically sterile or agreed to use double efficient contraceptive methods and who committed to avoid sperm donation for at least 6 months after the first administration of the drug. Exclusion criteria comprised any organic or psychic condition, previous use of prescription pharmacological treatment, body mass index (BMI) outside of the 18.5–30 kg/m^2^ range, consumption of abuse drugs, alcohol, or tobacco, blood donation in the previous month before starting the trial, and history of swallowing problems.

Clinical trial 1, with EUDRA-CT 2021-001334-19 (2021), and clinical trial 2, with EUDRA-CT 2021-001350-65 (2021), were included in the exploratory analysis. Both were bioequivalence, phase-I, crossover, open-label, randomized clinical trials of two formulations of tadalafil/finasteride 5 mg/5 mg film-coated tablets after administration of a single oral dose to male healthy volunteers.

Clinical trial 3, with EUDRA-CT 2013-001079-20 (2013), clinical trial 4, with EUDRA-CT 2014-001073-13 (2014), clinical trial 5, with EUDRA-CT 2015-000164-34 (2015), clinical trial 6, with EUDRA-CT 2015-000164-34 (2015), clinical trial 7, with EUDRA-CT 2016-001697-14 (2016), clinical trial 8, with EUDRA-CT 2022-000374-26 (2022), and clinical trial 9, with EUDRA-CT 2022-000357-81 (2022) were included in the confirmatory analysis. Clinical trials 3, 4, 5, 6, 7, 8, and 9 were bioequivalence, phase-I, crossover, open-label, randomized clinical trials of two formulations of tadalafil 20 mg after administration of a single oral dose to male (clinical trials 8 and 9) or male and female (clinical trials 3, 4, 5, 6, and 7) healthy volunteers. Only the reference tadalafil 20 mg film-coated tablets data was used for the confirmatory study. All clinical trials were only blinded for the analytical determination of tadalafil and finasteride plasma levels.

### 2.4. Description of Drug Used

Cialis^®^ 5 mg film-coated tablets (Eli Lilly Nederland B.V., Utrecht, The Nederland) and Proscar^®^ 5 mg film-coated tablets (Merck & Co., Inc., Rahway, NJ, USA) were administered as reference formulation (R) in clinical trials 1 and 2. Cialis^®^ 20 mg film-coated tablets (Eli Lilly Nederland B.V.) were administered in the remaining clinical trials. No information on the test formulation (T) can be provided from any clinical trial as this information is protected at the request of the sponsor of the clinical trials.

### 2.5. Feeding Conditions

Clinical trials 1 and 2 only differed in the feeding conditions when the drug was administered. Formulations were administered under fed conditions, after the administration of a 900-kcal high-fat breakfast according to EMA guidelines for bioequivalence clinical trials in one clinical trial and under fasting conditions in the other. Both had two periods and two sequences (TR and RT). In the first period, the volunteers were randomly administered the T or R formulations and, in the following period, after a 7-day washout period, the opposite formulation. In this way, it was ensured that each volunteer received each formulation twice. Volunteers were hospitalized from 10 h before to 12 h after dosing. A total of 72 volunteers participated in the bioequivalence clinical trials 1 and 2, 66 of whom gave informed consent to participate in the observational pharmacogenetic study. Healthy volunteers self-declared their biogeographic origin, biological sex, and age. Moreover, their weight and height were measured during the screening process to evaluate whether they met the inclusion criteria.

In clinical trials 3, 4, 5, 6, 7, 8, and 9, Cialis^®^ 20 mg film-coated tablets (Eli Lilly Nederland B.V.) were administered as R. No information on T can be provided as this information is protected at the request of the sponsors of the clinical trials. Clinical trials 3 and 4, 5, and 6, and 8 and 9 bioequivalence trials only differed in the feeding conditions when the drug was administered. Formulations were administered under fasting conditions in one clinical trial and under fed conditions after the administration of a high-fat 900-kcal breakfast according to EMA guidelines for bioequivalence clinical trials in the other. All clinical trials had two periods and two sequences (TR and RT). In the first period, the volunteers were randomly administered T or R formulations and, in the following period, after a 7-day washout period, the opposite formulation. In this way, it was ensured that each volunteer received each formulation twice. Volunteers were hospitalized from 10 h before to 12 h after dosing. Clinical trial 7 was a bioequivalence trial under fasting conditions with two periods and two sequences (TR and RT). In the first period, the volunteers were randomly administered the T or the R formulations and, in the following period, after a 7-day washout period, the opposite formulation. In this way, it was ensured that each volunteer received each formulation twice. Volunteers were hospitalized from 10 h before to 12 h after dosing. A total of 228 volunteers participated in the bioequivalence clinical trials 3, 4, 5, 6, 7, 8, and 9, 189 of whom provided informed consent to participate in the observational study. Healthy volunteers self-declared their biogeographic origin, biological sex, and age. Moreover, their weight and height were measured during the screening process to evaluate the inclusion criteria.

### 2.6. Pharmacokinetics Analysis

For pharmacokinetic profiling, clinical laboratory analyses and tadalafil and finasteride plasma level determinations were outsourced in both trials.

In each period, 22 blood samples were collected in 5-mL EDTA K2 tubes from each volunteer at different times between 0 h or predose (before receiving the drug) and up to 72 h after the administration of the drug. Immediately after blood collection, samples were centrifuged at 4 °C for 10 min at 1900 g and stored at −20 °C (±5 °C) until their shipment to an external laboratory. Drug determinations were performed after liquid-liquid extraction by high-performance liquid chromatography coupled with a tandem mass spectrometry detector (HPLC-MS/MS), with a lower limit of quantification (LLOQ) of 1 ng/mL for both drugs. The quantification method was performed according to the European legislation on bioequivalence clinical trials, the International Council for Harmonization of Technical Requirements for Pharmaceuticals for Human Use (ICH), and EMA guidelines [29,30].

The WinNonLin Professional version 8.3 software (Scientific Consulting, Inc., Cary, NC, USA) was used to calculate pharmacokinetic parameters following a non-compartmental model. The C_max_ and the t_max_ were obtained directly from the plasma concentration-time curves. The AUC between predose and the last time point at which the concentration was determined (AUC_0–t_) was calculated by the trapezoidal method. The remaining AUC from t to infinite (AUC_t–∞_) was calculated as the ratio C_t_/K_e_, where C_t_ is the last detectable concentration and K_e_ the elimination slope, obtained by linear regression of the log-linear part of the concentration–time curve. Furthermore, the total AUC (AUC_∞_) was determined by the sum of AUC_t–∞_ plus AUC_0–t_ and the t_1/2_ was calculated as ln2/K_e_. The T formulation proved to be bioequivalent to Cialis^®^ and Proscar^®^ in clinical trials 1 and 2, thus the arithmetic mean of the pharmacokinetic parameters was calculated for each volunteer for the exploratory study. Only Cialis^®^ data from clinical trials 3, 4, 5, 6, 7, 8, and 9 were used for the confirmatory clinical trial.

### 2.7. Genotyping

DNA extraction from peripheral blood was performed using either a MagNa Pure automatic DNA extractor (Roche Applied Science, Penzberg, Germany) or a Maxwell^®^ RSC instrument (Promega, Madison, WI, USA). DNA concentration was measured with a Qubit 3.0 Fluorometer (ThermoFisher, Waltham, MA, USA).

The genotyping was performed in a QuantStudio 12k Flex real-time PCR System (Applied Biosystems, ThermoFisher, Waltham, MA, USA) along with an Open Array thermal block and a customized array. It is based on quantitative polymerase chain reaction (qPCR) technology by allelic discrimination using TaqMan^®^ hydrolysis probes (Thermo Fisher Scientific, Waltham, MA, USA). Furthermore, a copy number variation (CNV) assay, which allows the identification of the gene deletion (*5) or gene duplications (xN), was performed in the same instrument with a 96-well thermal block for *CYP2D6* (assay IDs: Hs04083572_cn and Hs00010001_cn for intron 2 and exon 9, respectively; Thermo Fisher Scientific, Waltham, MA, USA) using RNAseP as the 2-copy reference.

Volunteers were genotyped for variants in genes potentially related to tadalafil/finasteride absorption, distribution, metabolism, and excretion based on clinically relevant pharmacogenes. Supplementary Table S1 shows the 120 variants and 33 genes analyzed for the present work. They were included for their potential relationship with pharmacokinetic variability of tadalafil or finasteride. The pharmacogene selection was based on the Very Important Pharmacogenes (VIP) from PharmGKB database [31]. Higher evidence variables, such as all enzymes and transporters with available pharmacogenetic phenotype, and lower evidence genes, such as *SLC22* family or *UGT* family, were included. All genotype changes are annotated according to RefSeq if available [32]. Due to the genotyping approach, not all individuals were successfully genotyped for all genes. Each table in the results section shows the total number of valid subjects for each case.

### 2.8. Haplotype Definition and Phenotype Inference

Genotypes were used to define haplotypes or alleles and to inform phenotypes. The pharmacogenetic phenotype was assigned according to Pharmacogene Variation Consortium (PharmVar) [33] core allele rules, Clinical Pharmacogenetics Implementation Consortium (CPIC) guidelines, and PharmGKB [31]. These guidelines provide information about what variants define star (*) alleles, the function of each allele, and the phenotype assigned to each diplotype. The following CPIC guidelines and allele definition tables were used: *CYP2B6* and efavirenz [34], *CYP2C19* and clopidogrel [35]; *CYP2D6* and atomoxetine [36]; *CYP3A5* and tacrolimus [37]; *SLCO1B1*, *ABCG2* and *CYP2C9* and statins [24]; *TPMT*, *NUDT15* and thiopurines [38]; and *UGT1A1* and atazanavir [39]. *CYP2C8* alleles were defined according to PharmVar database [33]. *NAT2* alleles were defined according to the Arylamine N-acetyltransferase Gene Nomenclature Committee [40]. Variants in the remaining genes without allele definitions or phenotype assignment were individually analyzed.

### 2.9. Statistical Analysis

The SPSS software version 29.0 (SPSS Inc., Chicago, IL, USA) and R software version 4.3.1 [41] were used for statistical analysis. The AUC_∞_ and C_max_ variables were adjusted for the dose/weight ratio (DW) in order to correct the impact of dose and weight on drug exposure. All pharmacokinetic parameters were logarithmically transformed to normalize distributions. The Shapiro–Wilk test, as well as the Q-Q plot, were used to verify the normality of the data set.

In the first place (exploratory study), a univariate analysis of the demographic characteristics according to the study design and biogeographic group was conducted in clinical trials 1 and 2, and of the pharmacokinetic parameters based on the study design, biogeographic group, genotypes, and phenotypes. For variables following normal distributions, means and standard deviations (SD) or coefficient of variation (CV%) were provided; for those not following normal distributions, medians, and the interquartile range (IQR, or the difference between the third and first quartile) were shown. *T*-tests were used for the comparison of means for variables with two categories and ANOVA tests for variables with three or more categories, followed by a Bonferroni post-hoc. When parametric tests were not applicable, a Mann–Whitney U test (two categories within a variable) or a Kruskal–Wallis one-way analysis of variance test (three or more categories within a variable) were used.

In second place (exploratory study), those factors with *p* < 0.05 in ANOVA or *t* test were introduced as independent variables in a multivariate analysis, which was performed by linear regression. As dependent variables, all pharmacokinetic parameters were explored. Variables with three or more groups were dummyfied following Bonferroni post hoc results. For the multivariate analysis, the significant relationships (*p*_MV_ < 0.05) were indicated with the unstandardized β-coefficient and R^2^ values. Type I error rate was set at α = 0.05 regardless of the type of analysis and the predetermined level of statistical significance at *p* < 0.05. A Bonferroni correction for multiple comparisons was performed in the multivariate analysis to control type I error.

In third place (confirmatory study), statistically significant variables for tadalafil were further analyzed in clinical trials 3 to 9 following the same statistical procedures as clinical trials 1 and 2.

## 3. Results

### 3.1. Exploratory Study

#### 3.1.1. Demographic Characteristics

A total of 66 healthy male volunteers consented to the pharmacogenetic study in clinical trials 1 and 2, with a mean age of 31.67 ± 8.47 years old, mean height of 1.73 ± 0.06 m, mean weight of 77.14 ± 9.95 kg, and body max index (BMI) of 25.62 ± 2.67 m/kg^2^.

The population was composed of 53 (80%) Latino-Americans and 13 (20%) European individuals. No significant differences in the demographic characteristics were observed based on biogeographic group (Table 1). Volunteers who participated in the clinical trial under fed conditions presented significantly higher age and BMI than volunteers who participated in the clinical trial under fasting conditions (*p* < 0.05 and *p* < 0.005, respectively), but no significant variation was observed for weight and height according to the study design (Table 1).

#### 3.1.2. Finasteride

Study population showed a mean AUC_∞_ (CV) of 260.55 (31.41%) ng·h/mL, C_max_ of 34.90 (24.79%) ng/mL, median (IQR) t_max_ of 2.34 (1.91) h and t_1/2_ of 5.68 (2.45) h. Fed volunteers presented a 1.2- and 1.8-fold higher AUC_∞_/DW (*p* = 0.032) and t_max_ (*p* < 0.001), respectively, compared to fasting volunteers, but not higher C_max_/DW (Table 2). No other pharmacokinetic association regarding the demographic characteristics of the study population was observed.

Regarding finasteride pharmacogenetics, variants in some genes (*CYP1A2*, *NUDT15*, *SLC22A1*, *SLC22A2*) were nominally associated with pharmacokinetic variation in finasteride, but these associations were lost after Bonferroni correction for multiple comparisons (Table 3).

*CYP3A4* did not show sufficient variability to be analyzed. Only one CYP3A4 *1/*3 intermediate metabolizer (IM) was present in the study. This volunteer presented AUC_∞_/DW of 4082.33 kg·ng·h/mL·mg, C_max_/DW of 471.88 kg·ng/mL·mg, t_max_ of 1 h, and t_1/2_ of 5.43 h.

#### 3.1.3. Tadalafil

The study population showed a mean (CV) AUC_∞_ of 2819.02 (43.79%) ng·h/mL, C_max_ of 111.36 (25.29%) ng/mL, median (IQR) t_max_ of 2.50 (2.48) h and t_1/2_ of 19.42 (9.67) h. Fed volunteers presented a 1.4-, 1.1, and 1.8-fold higher AUC_∞_/DW (*p* = 0.004), C_max_/DW (β = 0.122, R^2^ = 0.162, *p*_MV_ = 0.043), and t_max_ (*p* < 0.001), respectively, compared to fasting volunteers (Table 4). No other pharmacokinetic association was significant regarding the demographic characteristics of the study population.

Regarding tadalafil pharmacogenetics, variants in some genes (*ABCC3*, *CYP1A2*, *CES1*, *NUDT15*, *SLC22A1*, *UGT2B10*) were nominally associated with pharmacokinetic variation in tadalafil but did not remain significant after Bonferroni correction for multiple comparisons (Table 5).

*CYP3A4* did not show sufficient variability to be analyzed. Only one CYP3A4 *1/*3 intermediate metabolizer (IM) was present in the study. This volunteer presented AUC_∞_/DW of 29,088.21 kg·ng·h/mL·mg, C_max_/DW of 1544.65 kg·ng/mL·mg, t_max_ of 1 h, and t_1/2_ of 17.39 h.

### 3.2. Confirmatory Study

#### 3.2.1. Demographic Results

A total of 189 healthy volunteers consented to the confirmatory study in clinical trials 3, 4, 5, 6, 7, 8, and 9, with a mean age of 27.71 ± 7.74 years old, mean height of 70.45 ± 12.78 m, mean weight of 70.45 ± 12.78 kg, and body max index (BMI) of 24.18 ± 3.06 m/kg^2^. Only biogeographical group, study design, and sex were analyzed in the confirmatory analysis. The population was composed of 134 (71%) European individuals and 55 (29%) Latino-Americans. Latino-American volunteers presented significantly higher age, weight, and BMI than European volunteers (Table 6). The population also comprised 118 males (62%) and 72 females (38%). Males presented significantly higher age, weight, height, and BMI than females (Table 6).

No difference regarding demographic data was observed in individuals participating in the feeding conditions.

#### 3.2.2. Tadalafil

The study population showed a mean (CV) AUC_∞_ of 8568.77 (37.16%) ng·h/mL, C_max_ of 330.58 (31.49%) ng/mL, median (IQR) t_max_ of 3.00 (3.00) h and t_1/2_ of 20.28 (8.42) h.

**Figure 1:**
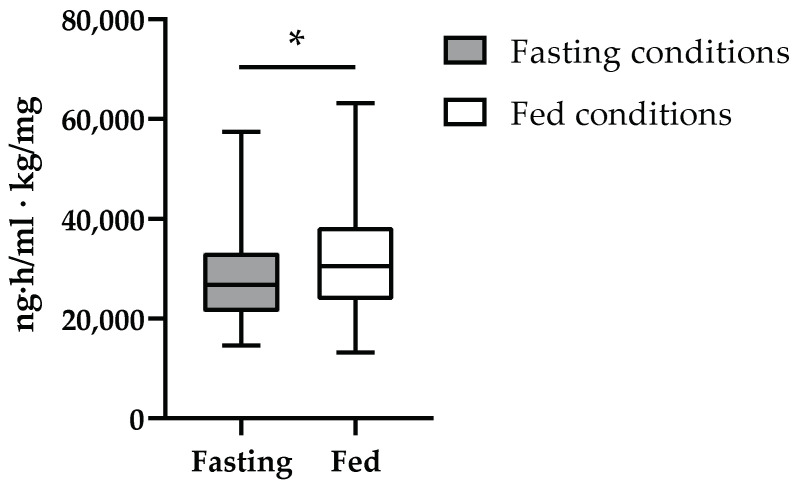
Tadalafil AUC_∞_/DW in clinical trials 3, 4, 5, 6, 7, 8, and 9 according to feeding conditions. * p < 0.05 after univariate and multivariate analysis.

Fed volunteers presented 1.2-, 1.2-, and 1.5-fold higher AUC_∞_/DW (β = 0.175, R^2^ = 0.113, *p*_MV_ = 0.001), C_max_/DW (β = 0.168, R^2^ = 0.170, *p*_MV_ < 0.001) and t_max_ (β = −0.132, *p*_MV_ < 0.001), respectively, compared to fasting volunteers (Table 7). Male volunteers presented 1.2- and 1.2-fold higher AUC_∞_/DW (β = 0.141, R^2^ = 0.113, *p*_MV_ = 0.009) (Figure 1) and C_max_/DW (β = 0.150, R^2^ = 0.168, *p*_MV_ < 0.001), respectively, compared to female volunteers (Table 7). No other pharmacokinetic association was significant regarding the demographic characteristics of the study population.

## 4. Discussion

Tadalafil pharmacokinetics in healthy individuals is linear in the dose range from 2.5 to 20 mg [8]. With a 20 mg dose, as reported in the literature, the AUC_∞_ and C_max_ values were 8066 ng·h/mL and 378 ng/mL, respectively; in our study, the observed values were 2819.02 ng·h/mL and 111.36 ng/mL for 5 mg dose, and 8568.77 ng·h/mL and 330.58 ng/mL for 20 mg dose. Hence, the observed tadalafil pharmacokinetic parameters were comparable to those previously reported [15,17,42]. Healthy volunteers also presented finasteride pharmacokinetic parameters in general congruent with the literature, e.g., AUC_∞_ of 260.55 ng·h/mL, C_max_ of 34.9 ng/mL, and t_max_ of 2.63 h, compared with 267–288 ng·h/mL, 38.1 ng/mL and 2 h, respectively [15,20,21]. No significant differences were found between biogeographic groups and any pharmacokinetic parameter for either drug.

No effect of food on the pharmacokinetics of tadalafil or finasteride is recorded in their drug label [8,43]. This would, therefore, be the first study to find an association between food intake and altered tadalafil and finasteride exposure. Previous bioequivalence clinical trials reported no differences in tadalafil pharmacokinetics between fed and fasted conditions [44,45]. Conversely, we observed that fed volunteers presented higher AUC_∞_, C_max_, and t_max_ compared to fasting volunteers in exploratory analysis, later confirmed in confirmatory analysis. The t_max_ increase is especially relevant for ED. Taking tadalafil before or after eating might result in an uncomfortable delay (of 1.5 h) of the maximum therapeutic effect, which may affect the sexual health of a man with ED. The studies addressing interactions of 5-α reductase inhibitors with food are scarce. Previous studies state that food tends to decrease finasteride C_max_ and delay t_max_, though these changes do not affect the total bioavailability of the drug, and thus, it may be taken with or without a meal [22,46]. Consistently, in this work, fed volunteers presented 1.8-fold higher t_max_ and 1.2-fold higher AUC_∞_ and Cmax than fasting volunteers, *p* value < 0.001.

Food and its constituents have a significant effect on the rate and extent of absorption of many drugs after oral administration [47]. Meals affect drug absorption by delaying gastric emptying time, altering gastrointestinal pH, stimulating bile flow, or physically interacting with drugs [48]. These interactions can be easily identified by observing absorption-specific pharmacokinetic parameters, such as t_max_ or C_max_ [49]. For tadalafil, food most likely delays gastric emptying, augmenting the transit time to the small intestine and, subsequently, delaying the absorption into the systemic circulation. As a result, the time to reach the maximum concentration (t_max_) increases, indicating a greater but slower gastric absorption in magnitude. This is consistent with previous studies that reported that the use of food slows down the effect of tadalafil [50]. Regarding finasteride, AUC_∞_ and t_max_ were also increased in fed volunteers. Therefore, the influence of meals on finasteride’s pharmacokinetics is likely to be comparable to that of tadalafil, with a potential delay in gastric emptying and subsequent effects on absorption. It is worth noting that this study evaluates the effect of food under ‘extreme’ conditions. However, a volunteer eating a high-fat meal does not necessarily reflect the circumstances of the patients who will take the drug. For instance, a higher-fat meal may produce a slower gastric emptying, potentially delaying t_max_.

Overall, understanding the impact of meals on drug absorption is crucial for optimizing treatment effectiveness [47]. Given these factors, clinicians can make informed decisions about drug administration, considering the potential influence of feeding conditions and adjusting dosage or timing accordingly. Further confirmatory studies are required to gain a comprehensive understanding of the impact of food on tadalafil and finasteride pharmacokinetics and to determine whether this effect has a clinically relevant impact on the efficacy and safety of treatment.

In addition, due to the indications for finasteride, sex could not be analyzed as a covariate in the exploratory study. Nonetheless, it was included in the confirmatory studies for tadalafil, showing a clear effect on the pharmacokinetics of the drug. Tadalafil is also used in women, since it is commonly prescribed for pulmonary hypertension management [49,50,51]. Therefore, it is crucial to dispose the pharmacokinetic data to potentially apply these drugs in female population. Our study showed that male volunteers presented 1.2-fold higher AUC_∞_ and C_max_ than female volunteers, which may have implications for the safety profile of the drug. Tadalafil has vasodilator properties, which produce a slight and transient decrease in blood pressure that enhances the hypotensive effect of nitrates [8]. These changes in blood pressure are not associated with an increase in ADRs and they are not believed to be clinically meaningful [52]. Still, physicians should assess the cardiovascular status of their patients before prescribing tadalafil, as well as provide information about the potential effects of food on its absorption.

No significant associations were found between genotypes or phenotypes and variability in tadalafil pharmacokinetics, although variants in some genes (*ABCC3*, *CYP1A2*, *CES1*, *NUDT15*, *SLC22A1*, *UGT2B10*) were nominally associated with tadalafil pharmacokinetic variation. Tadalafil has not been described as a substrate for any of these enzymes or transporters, but one study reported that tadalafil reversibly inhibits CYP1A2-mediated metabolism [53]. Metabolism of tadalafil mainly occurs through the cytochrome P450 3A (CYP3A4) oxidative process. Unfortunately, statistical analysis for *CYP3A4* genotype could not be conducted due to the lack of genetic variability. Out of the 66 volunteers, there was only one subject with CYP3A4 *1/*3 genotype (IM phenotype), who participated in clinical trial 1 (under fasting conditions). The IM volunteer presented similar finasteride AUC_∞_/DW, C_max_/DW, t_max_, and t_1/2_ to the mean of volunteers under the fasting conditions. Nonetheless, this IM volunteer showed a tendency for lower AUC_∞_/DW for tadalafil compared to the mean of volunteers under fasting conditions. Conversely, CYP3A4 reduced activity would increase tadalafil levels due to the reduced metabolism and elimination of the drug. Further research is needed in order to assess the effect of CYP3A4 polymorphisms and its pharmacokinetic impact on tadalafil.

Finasteride is extensively metabolized in the liver, involving *CYP3A4*-mediated hydroxylation and oxidation reactions [25]. There is a high degree of sequence homology between *CYP3A4* and *CYP3A5*, resulting in overlapping substrate specificity [37]. Consequently, it is anticipated that finasteride acts as a substrate for *CYP3A5*, and the *CYP3A5* phenotype contributes to its pharmacokinetic variability. In fact, a recent study showed an association between finasteride serum concentrations and genetic variations in the *CYP3A4* and *CYP3A5* genes [25]. The uridine diphosphoglucuronosyl transferase (UGT) enzymes also participate in finasteride metabolism, but there is little literature to suggest which UGT protein(s) are involved in its glucuronidation [26]. In the conducted study, no correlation was found between *CYP3A5* or *UGT* genotype and finasteride pharmacokinetic variability. However, variants in some genes (*CYP1A2*, *NUDT15*, *SLC22A1*, *SLC22A2*) were nominally associated with finasteride pharmacokinetic variation, although finasteride has not previously been reported to be a substrate of these enzymes or transporters.

The observed absence of clear genetic associations in the variability of tadalafil and finasteride pharmacokinetics may indicate that other factors, such as feeding conditions, may be more relevant in drug response. Further studies are required to confirm these findings.

### 4.1. Study Limitations

This study presents several limitations. The sample size is small, arbitrary, and was not calculated to demonstrate associations. Furthermore, the study population constituted a mixture of different biogeographical populations that do not represent a real population. This bias was controlled as the functional impact of the majority of the variants analyzed is known. Therefore, they are independent of biogeographical origin. In addition, a multivariate analysis was performed, including both biogeographic origin and the genetic/phenotypic variables. Also, the mixture of different bioequivalence clinical trials could introduce overlooked confounders. Therefore, the nature of our study is purely descriptive. It would be appropriate to increase the sample size in further confirmatory studies in order to acquire statistical power and to assess the impact of rare variants, such as *CYP3A4* *20 and *22. However, the confirmation of the food–drug interaction significantly increases the confidence for this association.

The lack of significant association in our study could be due to type 2 error or to the candidate-gene study design. Candidate gene studies are hypothesis-driven studies with high statistical power to identify gene–drug associations [51]. However, these studies miss the thousands of other genes and variants present in the genome, such as PD-related genetic variants or other yet unknown PK variants [54,55]. Hence, genome-wide association studies (GWAS) are crucial to identify potential associations and variants that may contribute to pharmacokinetic effects [55]. Similarly, genotypic inference from the genotyping of individual variants does not allow the identification of potential rare alleles that may occur in the population. Next Generation Sequencing (NGS) might be useful in additional studies to explore novel variants with a functional impact.

Lastly, the recruitment of healthy volunteers did not allow studying the efficacy and pharmacodynamics of tadalafil and finasteride. Although tadalafil-finasteride 5 mg/5 mg combination safety has been proved in clinical trials, the combination is not yet supported by American and European urological guidelines as data on this combination are considered emerging [56]. Further studies regarding tadalafil/finasteride combination safety are guaranteed. However, the study was performed in a very controlled environment that allowed confounding factors avoidance. This, together with the participation of single-sex volunteers, contributes to a significant reduction in pharmacokinetic variability.

### 4.2. Conclusions

The AUC_∞_, C_max_, and t_max_ of tadalafil and finasteride were delayed and increased as a consequence of food presence in the gastrointestinal tract. In addition, sex affects tadalafil pharmacokinetics, decreasing exposure in female volunteers. The clinical relevance of this interaction needs to be confirmed with additional studies. Tadalafil and finasteride pharmacokinetic parameters were unrelated to genetic variation. However, the impact of *CYP3A4* variation on drug pharmacokinetics needs to be further studied.

## Figures and Tables

**Table 1:** Demographic characteristics of study population in clinical trials 1 and 2 according to biogeographic group and study design.

	n	Age	Weight (kg)	Height (m)	BMI (kg/m^2^)
Total	66	31.67 ± 8.47	77.14 ± 9.95	1.73 ± 0.06	25.62 ± 2.67
Biogeographic group:					
Latino-American	53	31.32 ± 8.11	77.00 ± 10.73	1.73 ± 0.07	25.60 ± 2.84
European	13	33.08 ± 10.04	77.72 ± 6.10	1.74 ± 0.05	25.68 ± 1.93
Study design:					
Fasting conditions	33	29.55 ± 7.72	74.87 ± 8.50	1.74 ± 0.06	24.66 ± 2.44
Fed conditions	33	33.79 ± 8.77 *†	79.41 ± 9.95	1.73 ± 0.07	26.57 ± 2.57 *†

Results are shown as mean ± standard deviation. *: *p* < 0.05 after univariate analysis. †: *p*_multivariate (MV)_ < 0.05.

**Table 2:** Finasteride pharmacokinetic characteristics of study population by study design and biogeographic group of origin.

	n	AUC_∞_/DW	C_max_/DW	t_max_	t_1/2_
		(kg·ng·h/mL·mg)	(kg·ng/mL·mg)	(h)	(h)
Total	66	4018.84 (34.62%)	530.86 (21.01%)	2.34 (1.91)	5.68 (2.45)
Study design:					
Fasting conditions	33	3649.22 (30.18%)	544.93 (21.62%)	1.63 (1.33)	5.34 (2.54)
Fed conditions	33	4388.47 (35.57%) *	516.79 (20.26%)	3.00 (2.38) *	6.06 (2.58)
Biogeographic group:					
Latino-American	53	4037.34 (38.81%)	527.41 (21.18%)	2.34 (1.98)	5.75 (2.54)
European	13	3943.43 (24.14%)	544.93 (20.94%)	2.13 (1.52)	5.43 (2.07)

AUC_∞_/DW: area under the time-concentration curve from time 0 to infinity, corrected by dose/weight ratio; C_max_/DW: maximum drug concentration in plasma corrected by dose/weight ratio; t_max_: time to reach C_max_; t_1/2_: elimination half-life. AUC_∞_/DW and C_max_/DW data are shown as mean (coefficient of variation), t_max_ and t_1/2_ data are presented as median (Inter Quartile Range). *: *p*_MV_ < 0.05 after univariate analysis.

**Table 3:** Finasteride pharmacokinetic characteristics of study population by genotype or phenotype.

Gene	Genotype/Phenotype		n	AUC_∞_/DW	C_max_/DW	t_max_	t_1/2_
				(kg·ng·h/mL·mg)	(kg·ng/mL·mg)	(h)	(h)
*CYP1A2*	rs2069526	G/G	48	3934.41 (34.84%)	532.46 (21.66%)	2.04 (1.50)	5.41 * (2.55)
		G/T + T/T	6	4523.35 (27.06%)	575.88 (17.87%)	2.75 (0.75)	6.01 (1.16)
*NUDT15*	Phenotype	NM	61	3991.55 (35.91%)	522.32 (19.59%)	2.38 (1.94)	5.63 (2.50)
		IM	3	4367.35 (22.23%)	753.04 *† (2.41%)	2.34	5.75
*SLC22A1*	rs628031	C/C	30	4328.82 (31.02%)	588.76 *† (18.39%)	2.04 (1.42)	6.05 (1.68)
		C/A	23	3733.87 (34.43%)	484.81 (20.42%)	2.75 (1.88)	5.23 (3.58)
		A/A	4	3324.80 (19.53%)	494.70 (15.08%)	2.00 (1.00)	5.32 (2.22)
*SLC22A2*	rs316019	C/C	42	4205.07 (33.23%)	558.02 *† (19.03%)	2.11 (1.50)	6.00 (2.54)
		A/C + A/A	16	3438.34 (25.75%)	492.77 (24.04%)	2.38 (1.72)	5.25 (2.00)

AUC_∞_/DW: area under the time-concentration curve from time 0 to infinity, corrected by dose/weight ratio; C_max_/DW: maximum drug concentration in plasma corrected by dose/weight ratio; t_max_: time to reach C_max_; t_1/2_: elimination half-life; NM: normal metabolizers; IM: intermediate metabolizers; AUC_∞_/DW and C_max_/DW data are shown as mean (coefficient of variation), t_max_ and t_1/2_ data are shown as median (Inter Quartile Range). *: *p* < 0.05 after univariate analysis; †: *p*_MV_ < 0.05 after multivariate analysis. No significant results after Bonferroni correction for multiple comparisons. Variants are shown as annotated on RefSeq sequences when available.

**Table 4:** Tadalafil pharmacokinetic parameters according to the study design and biogeographic group of origin.

	n	AUC_∞_/DW	C_max_/DW	t_max_	t_1/2_
		(kg·ng·h/mL·mg)	(kg·ng/mL·mg)	(h)	(h)
Total	66	43,816.04 (46.66%)	1704.16 (26.43%)	2.50 (2.48)	19.42 (9.67)
Study design:					
Fasting conditions	33	37,102.48 (46.22%)	1595.92 (18.98%)	2.09 (1.61)	17.58 (8.08)
Fed conditions	33	50,529.59 (42.55%) *	1812.40 (30.03%) †	3.75 (3.38) *	20.52 (10.24)
Biogeographic group:					
Latino-American	53	44,093.50 (45.69%)	1693.89 (28.00%)	2.50 (2.56)	19.43 (10.29)
European	13	42,684.85 (38.14%)	1746.06 (19.83%)	2.88 (2.25)	19.42 (8.90)

AUC_∞_/DW: area under the time-concentration curve from time 0 to infinity, corrected by dose/weight ratio; C_max_/DW: maximum drug concentration in plasma corrected by dose/weight ratio; t_max_: time to reach C_max_; t_1/2_: elimination half-life. AUC_∞_/DW and C_max_/DW data are shown as mean (coefficient of variation), t_max_ and t_1/2_ data are presented as median (Inter Quartile Range). * *p* < 0.05 after univariate analysis. †: *p*_MV_ < 0.05 after multivariate analysis.

**Table 5:** Tadalafil pharmacokinetic characteristics of study population by genotype or phenotype.

Gene	Genotype/Phenotype		n	AUC_∞_/DW	C_max_/DW	t_max_	t_1/2_
				(kg·ng·h/mL·mg)	(kg·ng/mL·mg)	(h)	(h)
*ABCC3*	rs4793665	C/C	11	38,005.89 (44.28%)	1747.45 (21.53%)	1.88 (1.38)	19.97 (07.25)
		C/T	26	48,130.33 (38.61%)	1733.22 (18.20%)	3.48 * (2.78)	20.76 (11.03)
		T/T	21	41,104.26 (50.25%)	1695.94 (37.96%)	2.13 (1.96)	18.62 (11.92)
*CYP1A2*	rs2069526	G/G	48	43,099.64 (46.99%)	1709.75 (28.31%)	2.29 * (1.98)	19.42 (11.00)
		G/T + T/T	6	51,511.42 (27.42%)	1806.88 (21.71%)	4.31 (3.06)	21.54 (09.66)
*CES1*	rs2244613	T/T	37	41,577.13 (39.79%)	1748.40 (20.26%)	2.34 (1.67)	20.21 (10.55)
		G/T	16	41,148.53 (47.55%)	1530.29 *† (17.29%)	3.94 (3.85)	17.31 (09.51)
*NUDT15*	Phenotype	NM	61	42,792.27 (45.83%)	1672.15 (20.90%)	2.63 (2.44)	19.43 (09.64)
		IM	3	65,157.54 (51.94%)	2537.41 * (52.29%)	1.09 *	18.62
*SLC22A1*	rs72552763	GAT/GAT	43	39,931.85 (40.54%)	1647.64 (21.98%)	2.75 (1.75)	19.42 (08.03)
		-/GAT	15	59,170.04 * (43.81%)	1915.16 (34.79%)	2.13 (3.88)	22.32 (21.75)
		-/-	8	35,904.79 * (47.92%)	1612.39 (18.04%)	1.81 (4.04)	15.39 (08.72)
	rs12208357	C/C	64	44,475.26 (45.90%)	1709.94 (26.56%)	2.56 (2.59)	19.70 (09.83)
		C/T	2	22,720.83 (6.24%)	1519.10 (23.34%)	1.40	11.28 *
*UGT2B10*	rs6175090	G/G	48	41,943.58 (41.92%)	1679.04 (20.89%)	2.69 (2.31)	19.42 (09.54)
		G/T	5	35,061.77 (33.08%)	1589.25 (19.36%)	1.88 * (1.37)	19.97 (15.98)

AUC_∞_/DW: area under the time-concentration curve from time 0 to infinity, corrected by dose/weight ratio; C_max_/DW: maximum drug concentration in plasma corrected by dose/weight ratio; t_max_: time to reach C_max_; t_1/2_: elimination half-life; NM: normal metabolizers; IM: intermediate metabolizers; AUC_∞_/DW and C_max_/DW data are shown as mean (coefficient of variation), t_max_ and t_1/2_ data are shown as median (Inter Quartile Range). *: *p* < 0.05 after univariate analysis; †: *p*_MV_ < 0.05 after multivariate analysis. No significant associations were observed after Bonferroni correction for multiple comparisons. Variants are shown as annotated on RefSeq sequences when available.

**Table 6:** Demographic characteristics of study population in clinical trials 3, 4, 5, 6, 7, 8, and 9 according to biogeographic group, study design, and sex.

	n	Age	Weight (kg)	Height (m)	BMI (kg/m^2^)
Total	189	27.71 ± 7.74	70.45 ± 12.78	1.70 ± 0.09	24.18 ± 3.06
Biogeographic group:					
Latino-American	55	31.52 ± 8.28 *	74.28 ± 11.94 *	1.70 ± 0.09	25.52 ± 2.87 *
European	134	26.02 ± 6.94	68.94 ± 12.85	1.70 ± 0.08	23.61 ± 2.98
Study design:					
Fasting conditions	113	27.50 ± 7.81	70.52 ± 13.42	1.70 ± 0.09	24.20 ± 3.08
Fed conditions	77	27.75 ± 7.68	70.39 ± 11.83	1.70 ± 0.08	24.16 ± 3.04
Sex:					
Male	118	28.35 ± 7.56	77.18 ± 10.38	1.75 ± 0.06	25.14 ± 2.77
Female	72	26.39 ± 7.93 *	59.47 ± 7.67 *	1.62 ± 0.06 *	22.60 ± 2.87 *

Results are shown as mean ± standard deviation. *: *p* < 0.05 after univariate analysis.

**Table 7:** Tadalafil pharmacokinetic parameters in clinical trials 3, 4, 5, 6, 7, 8, and 9 according to the study design, biogeographic group of origin, and sex.

	n	AUC_∞_/DW	C_max_/DW	t_max_	t_1/2_
		(kg·ng·h/mL·mg)	(kg·ng/mL·mg)	(h)	(h)
Total	189	30,055.28 (37.16%)	1136.01 (31.49%)	3.00 (3.00)	20.28 (8.42)
Biogeographic group:					
Latino-American	55	32,264.37 (31.92%)	1265.604 (32.65%)	3.50 (3.33)	21.77 (7.93)
European	134	29,187.95 (39.24%)	1095.88 (30.35%)	3.00 (3.50)	19.54 (8.96)
Study design:					
Fasting conditions	113	27,740.29 (32.42%)	1036.88 (29.08%)	2.67 (2.55)	21.37 (8.18)
Fed conditions	77	33,453.41 (39.12%) *†	1251.78 (30.72%) *†	4.00 (2.90) *†	19.54 (7.81)
Sex:					
Male	118	31,935.53 (36.83%)	1219.41 (31.12%)	3.50 (2.69)	20.28 (8.97)
Female	72	26,969.32 (34.85%) *†	1024.61 (28.66%) *†	2.75 (3.00) *	20.34 (7.78)

AUC_∞_/DW: area under the time-concentration curve from time 0 to infinity, corrected by dose/weight ratio; C_max_/DW: maximum drug concentration in plasma corrected by dose/weight ratio; t_max_: time to reach C_max_; t_1/2_: elimination half-life. AUC_∞_/DW and C_max_/DW data are shown as mean (coefficient of variation), t_max_ and t_1/2_ data are presented as median (Inter Quartile Range). * *p* < 0.05 after univariate analysis. †: *p*_MV_ < 0.05 after multivariate analysis.

## Data Availability

Data would be available upon reasonable request.

## Supplementary Materials

The following supporting information can be downloaded at: https://www.mdpi.com/article/10.3390/jpm13111566/s1, Table S1: Genes, alleles and SNPs analyzed.
