# Supplementary material for: Food Administration and Not Genetic Variants Causes Pharmacokinetic Variability of Tadalafil and Finasteride
Source: J Pers Med. 2023 Oct 31;13(11):1566. doi: 10.3390/jpm13111566 (PMC10672114; doi:10.3390/jpm13111566)
Supplement: Supplementary file 1 [file jpm-13-01566-s001.zip › jpm-2641503-supplementary.pdf]

Supplementary Table S1. Genes, alleles and SNPs analyzed.

Gene	Alleles	SNP	Nucleotide change
ABCB1		rs1045642	T>C
		rs1128503	T>C
		rs2032582	T>G
			T>A
ABCC2		rs2273697	G>A
		rs3740066	C>T
ABCC3		rs4793665	C>T
		rs9895420	T>A
ABCG2		rs2231137	G>A
		rs2231142	C>A
		rs7699188	C>T
CES1		rs2244613	C>A
		rs8192935	T>C
		rs71647871	G>A
CYP1A1		rs1048943	A>G
		rs1799814	C>A
		rs4646903	T>C
CYP1A2		rs762551	C>A
		rs2069514	G>A
		rs2069526	T>G
		rs2470890	T>C
		rs12720461	C>T
		rs72547516	A>T
CYP1B1		rs10012	C>G
		rs1056836	C>G
		rs1800440	A>G
CYP2A6		rs28399433	T>G
CYP2B6	*4, *5, *6, *7, *9, *18, *22, *34, *36	rs2279343	A>G
		rs3211371	C>T
		rs3745274	G>T
		rs34223104	T>C
		rs28399499	T>C
CYP2C8	*2, *3, *4	rs1058930	C>G
		rs10509681	A>G
		rs11572080	G>A
		rs11572103	A>T
CYP2C9	*2, *3, *5, *8, *11	rs1057910	A>C
		rs1799853	C>T
		rs7900194	G>T
		rs28371685	C>T
		rs28371686	C>G
CYP2C19		rs4244285	G>A

	*2, *3, *4, *5 ,*6, *7, *8, *9, *17, *35	rs12769205	A>G
		rs4986893	G>A
		rs28399504	A>G
		rs56337013	C>T
		rs72552267	G>A
		rs72558186	T>A
		rs41291556	T>C
		rs17884712	G>A
		rs12248560	C>T
CYP2D6	*2, *3, *4, *5, *6, *7, *8, *9, *10, *12, *14, *15, *17, *19, *29, *41, *56B, *59	rs16947	C>T
		rs1065852	C>T
		rs1135840	G>C
		rs3892097	G>A
		rs5030655	A>delA
		rs5030656	TCT>delTCT
		rs5030862	G>A
		rs5030865	G>A
		rs5030865	G>T
		rs5030867	A>C
		rs28371706	C>T
		rs28371725	G>A
		rs35742686	T>delT
		rs59421388	G>A
		rs72549347	C>T
		rs72549353	AGTT>delAGTT
		rs79292917	G>A
		rs774671100	A>dupA
CYP3A4		rs2242480	G>A
	*2, *3, *4, *5, *6, *18, *20, *22, *37	rs2740574	G>A
		rs55785340	T>C
		rs67666821	T>dupT
		rs4986910	T>C
		rs55951658	A>G
		rs55901263	C>G
		rs4646438	T>dupT
		rs28371759	T>C
		rs35599367	C>T
CYP3A43	*2	rs61469810	A>delA
CYP3A5	*3, *6, *7	rs776746	A>G
		rs10264272	G>A
		rs41303343	A>dupA
NAT2	*5, *6, *7	rs1799930	G>A
		rs1799931	G>A
		rs1801280	T>C
NUDT15	*3	rs116855232	C>T
SLC22A1		rs628031	A>G
		rs72552763	GAT>-

		rs12208357	C>T
		rs34059508	G>A
SLC22A2		rs316019	A>C
SLC28A3		rs7853758	G>A
SLC6A2		rs3785143	C>T
		rs12708954	C>A
SLCO1B1		rs4149015	G>A
	*1, *4, *5, *6, *9, *14, *15, *19, *20, *23,*31, *37,	rs2306283	A>G
		rs4149056	T>C
		rs11045819	C>A
		rs55901008	T>C
		rs56061388	T>C
		rs56101265	T>C
		rs56199088	A>G
		rs59502379	G>C
TPMT	*2, *3A, *3B, *3C, *4, *11	rs72552738	G>A
		rs1800462	G>C
		rs1800460	G>A
		rs1142345	A>C
		rs1800584	G>A
UGT1A1		rs8330	G>C
		rs10929302	G>A
	*6, *80	rs887829	C>T
		rs4148323	G>A
UGT1A3		rs2008584	A>G
UGT1A4		rs2011425	T>G
UGT1A6		rs7592281	G>T
		rs10445704	G>A
UGT1A8		rs1042597	C>G
UGT2B10		rs61750900	G>T
UGT2B15		rs1902023	T>G
UGT2B7		rs7668258	T>C

Nucleotide change reference is RefSeq except from *ABCC3*, *SLC22A1*, *SLC22A2*, *SLC28A3*, *UGT2B10* and *UGT2B7*, which is GRCh38 build.
